# Crystal structure of *catena*-poly[[tetra­aquamangan­ese(II)]-μ-1,5-dihy­droxynaphthalene-2,6-di­carboxyl­ato]

**DOI:** 10.1107/S205698902600040X

**Published:** 2026-01-20

**Authors:** Hitoshi Kumagai, Nobuhiro Ogihara

**Affiliations:** a Toyota Central R&D Labs., Inc., 41-1 Yokomichi, Nagakute, Aichi 480-1192, Japan; Tokyo University of Science, Japan

**Keywords:** crystal structure, manganese, hydrogen bonding, π–π inter­action, dihy­droxy-naphthalenedi­carboxyl­ate

## Abstract

The title compound, [Mn(H_2_dondc)(H_2_O)_4_]_*n*_ or [Mn(C_12_H_6_O_6_)(H_2_O)_4_]_*n*_, was synthesized by the reaction of manganese(II) chloride (MnCl_2_), 1,5-dihy­droxynaphthalene-2,6-di­carb­oxy­lic acid (H_4_dondc) and lithium hydroxide (LiOH). The asymmetric unit comprises half of an Mn^II^ ion, half of a 1,5-dihy­droxynaphthalene-2,6-di­carboxyl­ate dianion (H_2_dondc^2−^) and two water mol­ecules. The Mn^II^ ion is located on a crystallographic inversion center and exhibits a six-coordinated MnO_6_ octa­hedral geometry. The octa­hedron is comprised of two oxygen atoms from the two H_2_dondc^2−^ ligands and four oxygen atoms from the water mol­ecules. The carboxyl­ate group and naphthalene moiety lie almost coplanar to each other and show a monodentate coordination to the Mn^II^ ion. The planar H_2_dondc^2−^ ligands bridge Mn^II^ ions to form a one-dimensional chain along the diagonal direction of the *b* and *c* axes. In the crystal, there are two types of intra-chain hydrogen-bonding inter­actions. The first is between the phenolic hydroxyl groups and carboxyl­ate groups. The phenolic hydroxyl groups of the ligand are protonated and act as intra-chain hydrogen-bonding donors to coordinated oxygen atoms of the carboxyl­ate groups. The other is between coordinated water mol­ecules and non-coordinated oxygen atoms of the carboxyl­ate groups. The parallel chains are connected not only by inter-chain hydrogen-bonding inter­actions between coordinated water mol­ecules and phenolic hydroxyl groups but also by inter-chain hydrogen-bonding inter­actions between coordinated water mol­ecules to give two-dimensional networks. The chains are further connected by inter-chain π–π stacking inter­actions between the naphthalene moieties.

## Abstract 2

The Mn^II^ ions in the title compound are bridged by the H_2_dondc^2−^ (H_2_dondc^2−^ is 1,5-dihy­droxynaphthalene-2,6-di­carboxyl­ate) ligand to form a one-dimensional chain. The chains are further connected by π–π inter­actions, forming a three-dimensional network.

## 1. Chemical context

Metal–organic frameworks (MOFs) or coordination polymers (CPs) are compounds composed of metal ions and organic ligands that are connected by coordination bonds and form networks of different dimensionalities (one-dimensional, two-dimensional or three-dimensiona) in a crystal structure. These materials have received significant attention due to their diverse structures, and their physical and chemical applications, including magnetism, conductivity, gas sorption and catalytic activity (Kurmoo, 2009 ▸; Kurmoo *et al.*, 1995 ▸; Zhong *et al.*, 2023 ▸; Nakatani *et al.*, 1990 ▸; Kitagawa *et al.*, 2004 ▸). Multitopic organic ligands such as polypyridines, polyamines and polycarboxyl­ates are often used in the synthesis of these materials. Among these ligands, the benzene­dicarboxyl­ate ligand (bdc^2−^ dianion) and its analogues are well-known bridging ligands that yield functional materials (Kurmoo, 2009 ▸; Furukawa *et al.*, 2010 ▸). We have reported on electrode materials that use bdc^2−^ dianion analogues (Ogihara *et al.*, 2014 ▸, 2021 ▸, 2023 ▸; Yasuda & Ogihara, 2014 ▸) and magnetic materials that involve polycarboxyl­ate in which the number of carboxyl­ate groups and the distances between carboxyl­ate groups systematically vary (Kumagai *et al.*, 2001 ▸, 2002 ▸; Kurmoo *et al.*, 2001 ▸, 2003 ▸). We also reported a series of two-dimensional (2D) layered compounds that employ *R*_4_-benzene­dicarboxyl­ate (*R*_4_-bdc^2−^; *R* = H, F, Cl, and Br) as a bridging ligand and *M*^II^ ions (*M*^II^ = Mn, Co, Zn, and Cu). The structures and water adsorption-desorption properties are tuned by altering the halogen atoms attached to the benzene ring or metal ions used in these compounds (Kumagai *et al.*, 2012 ▸, 2021 ▸). 1,5-Dihy­droxynaphthalene-2,6-di­carb­oxy­lic acid (H_4_dondc) is also a di­carboxyl­ate analogue, in which phenolic hydroxyl groups are introduced within the naphthalene backbone. The H_4_dondc ligand can give four available charges (1− to 4−) depending on the deprotonation state, and MOFs with the 4− state of the ligand, where both the carboxyl groups and phenolic hydroxyl groups are deprotonated, were synthesized at high temperature using solvothermal reactions or microwaves to give honeycomb-type pores with open metal sites (Yeon *et al.*, 2015 ▸; Dietzel *et al.*, 2020 ▸). We have previously reported the first structural characterization of {[Co(H_2_dondc)(H_2_O)_4_]·2DMF}_*n*_ (DMF = *N*,*N*′-di­methyl­formamide, CCDC reference: 2421049, RURSIK), in which the ligand acts as a 2− anion (Kumagai *et al.*, 2025 ▸). Here, we have focused on the use of H_2_dondc^2−^ in the synthesis of an Mn^II^–H_2_dondc^2−^ dianion system under ambient conditions and report on the single-crystal structure of [Mn(H_2_dondc)(H_2_O)_4_]_*n*_.
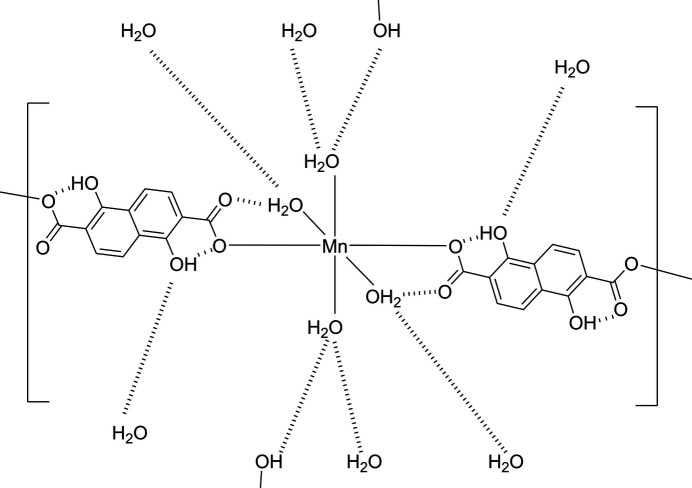


## 2. Structural commentary

**Figure 1:**
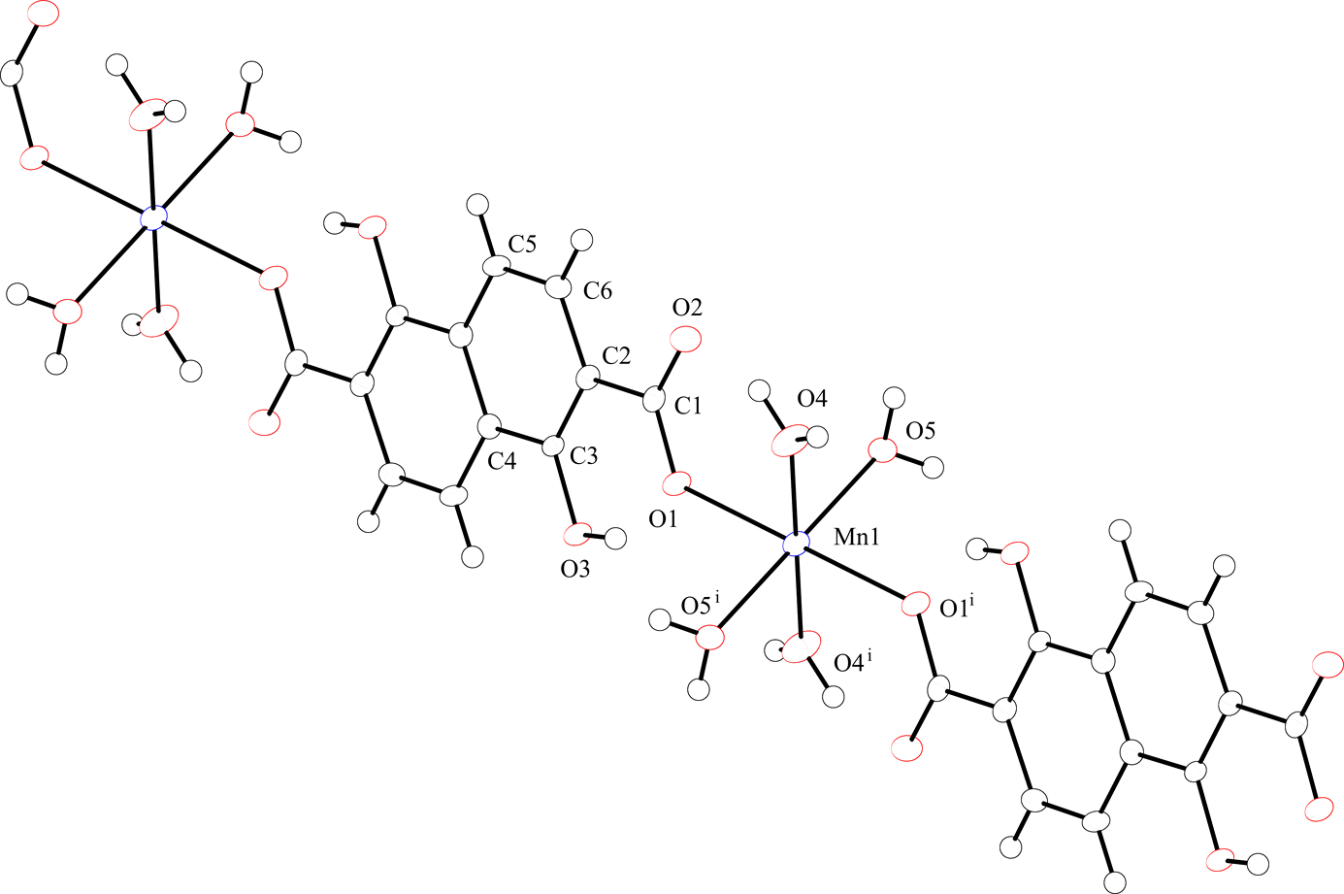
The one-dimensional chain structure of the title compound with the atom-labeling scheme and 50% probability displacement ellipsoids. Hydrogen atoms are omitted for clarity. [Symmetry code: (i) −*x* + 1, −*y* + 2, −*z* + 2.]

The title compound, [[Mn(H_2_dondc)(H_2_O)_4_]_*n*_, consists of an Mn^II^ ion, H_2_dondc^2−^ and four water mol­ecules. The Mn^II^ ion lies on a crystallographic inversion center and its asymmetric unit consists of half of an Mn^II^ ion, half of a H_2_dondc^2−^ ligand and two water mol­ecules. The characteristic point of the structure is a three-dimensional (3D) hydrogen-bonding network that consists of one-dimensional (1D) coordination chains built up by MnO_6_ octa­hedra bridged by H_2_dondc^2−^ ligands and inter-chain O—H⋯O hydrogen bonding and π–π stacking inter­actions of the naphthalene moieties. We have reported a similar compound, {[Co(H_2_dondc)(H_2_O)_4_]·2DMF}_*n*_, in which DMF (dimethylformamide) mol­ecules are included in the crystal (Kumagai *et al.*, 2025 ▸). Here we describe the structure of [Mn(H_2_dondc)(H_2_O)_4_]_*n*_ and the differences between this structure and {[Co(H_2_dondc)(H_2_O)_4_]·2DMF}_*n*_. Comparisons of selected bond distances, angles and hydrogen-bonding geometry are summarized in the supporting information. Fig. 1 ▸ shows the one-dimensional chain structure of [Mn(H_2_dondc)(H_2_O)_4_]_*n*_ with the numbering scheme. The Mn^II^ ion occupies a crystallographic inversion center; therefore, octa­hedron is formed and each pair of H_2_dondc^2−^ ligands and water mol­ecules coordinate *trans* positions to each other. The Mn—O1 (carboxyl­ate) bond length [2.1310 (13) Å] in [Mn(H_2_dondc)(H_2_O)_4_] is shorter than the Mn—O4 and Mn—O5 (H_2_O) bond lengths [2.1521 (16) Å and 2.2335 (15) Å, respectively], which is indicative of a slightly elongated octa­hedral geometry along the Mn—O5 bond. The ligands bridge the octa­hedral Mn^II^ ions to form a linear chain along the diagonal direction of the *b* and *c* axes. The Mn⋯Mn separation defined by Mn–H_2_dondc^2−^–Mn connectivity within the chain is 13.22 (5) Å, which is similar to that for the Co compound [13.27 (3) Å; Kumagai *et al.*, 2025 ▸). The carboxyl­ate group exhibits a monodentate coordination to the Mn^II^ ion and the phenolic hydroxyl groups show no coordination bonding to the Mn^II^ ion, giving the 2− anion. The phenolic hydroxyl groups show intra-chain hydrogen-bonding inter­actions with the coordinated oxygen atoms of the carboxyl­ate groups. The non-coordinated oxygen atom of the carboxyl­ate group (O2) shows intra-chain hydrogen-bonding inter­actions with coordinated water mol­ecules (O4) at an O⋯O distance of 2.756 (2) Å. The oxygen atoms of the carboxyl­ate groups act as intra-chain hydrogen-bond acceptors, and the coordinated water mol­ecules and phenolic hydroxyl groups act as intra-chain hydrogen-bond donors. These structural features are similar to those of the previously reported Co^II^ compound (Kumagai *et al.*, 2025 ▸). The difference between the structures of the title and Co^II^ compounds is the planarity between the carboxyl­ate group and naphthalene ring. While the carboxyl­ate group and the naphthalene ring are almost coplanar with an C6—C2—C1—O1 torsion angle of 179.77 (17)° in [Mn(H_2_dondc)(H_2_O)_4_]_*n*_, the Co^II^ compound shows a slightly tilted geometry with a torsion angle of 171.94 (11)°.

## 3. Supra­molecular features

**Figure 2:**
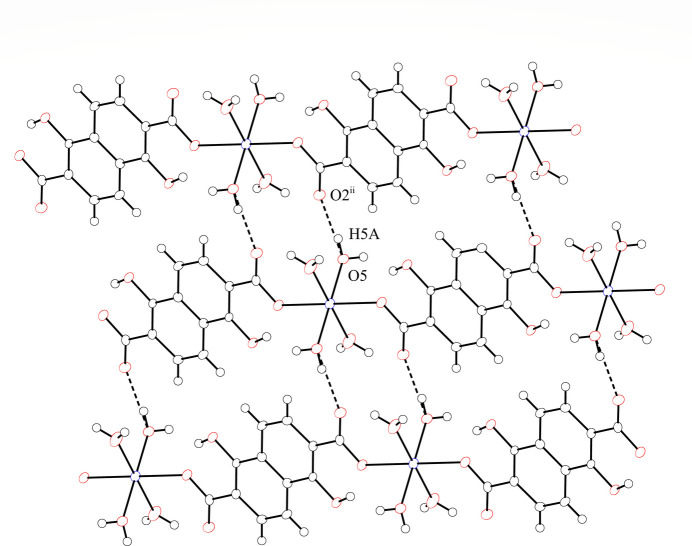
View of the two-dimensional hydrogen-bonding network in the planar direction of the naphthalene rings. Hydrogen-bonding inter­actions are shown as dashed lines.

**Figure 3:**
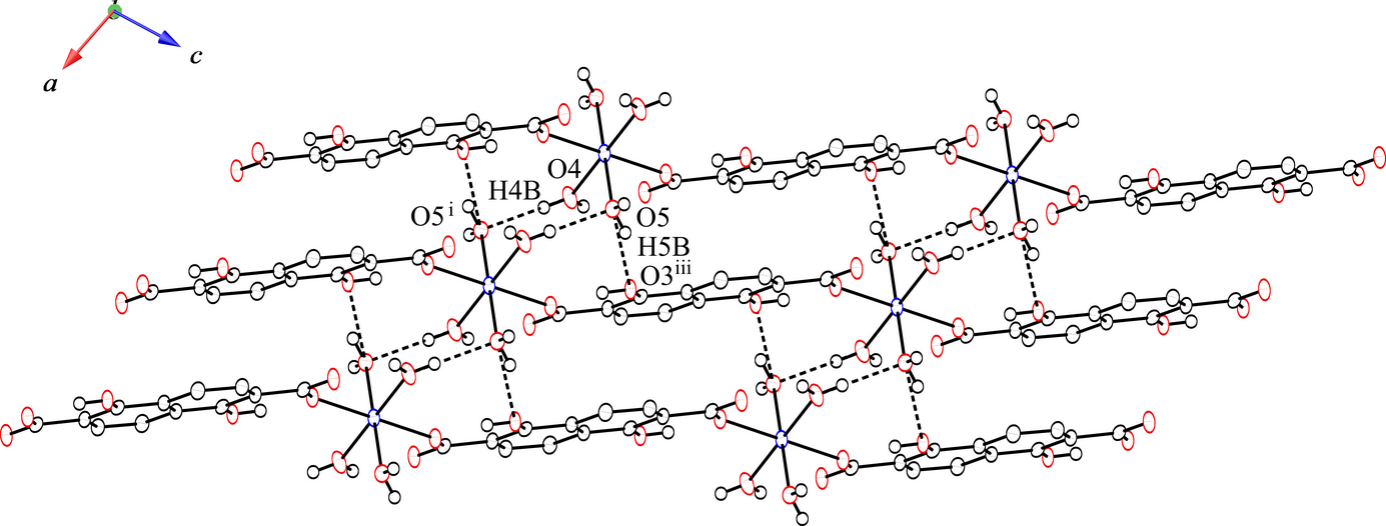
View of the two-dimensional hydrogen-bonding network in the direction of the naphthalene ring stacking. Hydrogen bonds are shown as dashed lines. Hydrogen atoms of the naphthalene rings are omitted for clarity.

The coordinated water mol­ecules of the title compound play important roles in forming intra- and inter-chain hydrogen-bonding inter­actions that yield a hydrogen-bonding network in the crystal structure (Table 1 ▸). The chains are hydrogen bonded both in the direction of naphthalene ring stacking and in the planar direction of the naphthalene rings to form a three-dimensional network. Fig. 2 ▸ shows the two-dimensional inter-chain hydrogen-bonding network in the planar direction of the naphthalene rings. The non-coordinated oxygen atoms (O2^ii^) of the carboxyl­ate groups hydrogen bond to coordinated water mol­ecules (O5) of the adjacent chain at a distance of 2.664 (2) Å. O2 acts as a hydrogen-bond acceptor and the coordinated water mol­ecule acts as a hydrogen-bond donor. This two-dimensional hydrogen-bonding network is similar to that of the previously reported Co^II^ complex (Kumagai *et al.*, 2025 ▸). Coordinated water mol­ecules (O4) show inter-chain hydrogen-bonding inter­actions at a distance of 2.814 (2) Å between the coordinated water mol­ecules of adjacent chains (O5) in the direction of naphthalene ring stacking, as shown in Fig. 3 ▸. The water mol­ecules also act as hydrogen-bond donors (O4) and as hydrogen-bond acceptors (O5). The coordinated water mol­ecules (O5) form two types of inter-chain hydrogen-bonding inter­actions in the direction of the naphthalene ring stacking. One is a hydrogen-bonding inter­action with a water mol­ecule of an adjacent chain [O4⋯O5^i^ = 2.814 (2) Å; symmetry code as in Table 1 ▸] and the other is a hydrogen bond with a phenolic hydroxyl group in a neighboring chain [O5⋯O3^iii^ = 2.798 (2) Å; symmetry code as in Table 1 ▸]. Water mol­ecules (O5) act not only as hydrogen-bond acceptors toward other water mol­ecules but also as hydrogen-bond donors to phenolic hydroxyl groups. The almost planar naphthalene moieties are stacked along the crystallographic *a*-axis direction in the crystal, with shortest centroid⋯centroid distances between the naphthalene rings and C⋯C distances of 3.7345 (13) and 3.378 (3) Å, respectively. These distances are indicative of π–π stacking inter­actions between the naphthalene moieties. The one-dimensional chains thereby form a three-dimensional network through hydrogen bonding and π–π stacking inter­actions. The difference between [Mn(H_2_dondc)(H_2_O)_4_]_*n*_ and the Co^II^ compound is the absence of DMF mol­ecules between the chains in the crystal structure (Kumagai *et al.*, 2025 ▸). The presence of DMF mol­ecules between the chains prevents π–π stacking between the naphthalene moieties; the naphthalene moieties of the Co^II^ compound showed C—H⋯π inter­actions between DMF mol­ecules and the naphthalene rings rather than the π–π stacking observed in the title compound. This result suggests that the inter-chain inter­actions can be controlled by the solvent in the crystal.

## 4. Database survey

We have previously reported the structure of {[Co(H_2_dondc)(H_2_O)_4_]·2DMF}_*n*_ and a database survey using the Sci Finder database and Web of Science concerning H_2_dondc^2−^ and Co^II^ (Kumagai *et al.*, 2025 ▸). This time a similar survey for H_2_dondc^2−^ and Mn^II^ ion was conducted that resulted in no complete matches. The structures of metal complexes composed of an Mn^II^ ion and a dondc^4−^ ligand that form a three-dimensional network consisting of hexa­gonal channels have been reported (CADYOZ and CADYUF; Dietzel *et al.*, 2020 ▸).

## 5. Synthesis and crystallization

Manganese(II) chloride hexa­hydrate (0.39 g, 2.00 mmol) was dissolved in ethanol (20 mL). Lithium hydroxide (0.09 g, 4.00 mmol) and H_4_dondc (0.24 g, 2.00 mmol) were dissolved in a mixture of water (10 mL) and DMF (10 mL). The Mn^II^ solution was poured into the mixture without stirring at room temperature. Single crystals were formed not only the interface of the solutions but also elsewhere due to the gradual diffusion of the solutions. Yellow crystals were obtained and one of these crystals was used for single-crystal X-ray crystallography analysis.

## 6. Refinement

The crystal data, data collection, and structure refinement details are summarized in Table 2 ▸. Hydrogen atoms attached to the phenolic hy­droxy group and water mol­ecules were extracted from difference-Fourier maps and refined isotropically. Other hydrogen atoms were placed in idealized positions (C—H = 0.95 Å) and refined using a riding model with *U*_iso_(H) = 1.2*U*_eq_(C).

## Supplementary Material

Crystal structure: contains datablock(s) I. DOI: 10.1107/S205698902600040X/jp2024sup1.cif

Structure factors: contains datablock(s) I. DOI: 10.1107/S205698902600040X/jp2024Isup2.hkl

Supporting information file. DOI: 10.1107/S205698902600040X/jp2024Isup4.cdx

Supporting information file. DOI: 10.1107/S205698902600040X/jp2024sup3.pdf

CCDC reference: 2523005

Additional supporting information:  crystallographic information; 3D view; checkCIF report

## Figures and Tables

**Table 1:** Hydrogen-bond geometry (Å, °)

*D*—H⋯*A*	*D*—H	H⋯*A*	*D*⋯*A*	*D*—H⋯*A*
O4—H4*A*⋯O2	0.88 (3)	2.02 (3)	2.756 (2)	141 (3)
O4—H4*B*⋯O5^i^	0.79 (3)	2.04 (3)	2.814 (2)	169 (3)
O3—H3⋯O1	0.79 (3)	1.75 (3)	2.480 (2)	153 (3)
O5—H5*A*⋯O2^ii^	0.80 (3)	1.88 (3)	2.664 (2)	168 (3)
O5—H5*B*⋯O3^iii^	0.71 (3)	2.13 (3)	2.798 (2)	157 (3)

Symmetry codes: (i) 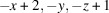; (ii) 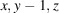; (iii) 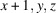.

**Table 2:** Experimental details

Crystal data	
Chemical formula	[Mn(C_12_H_6_O_6_)(H_2_O)_4_]
*M*_r_	373.17
Crystal system, space group	Triclinic, *P* 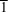
Temperature (K)	150
*a*, *b*, *c* (Å)	5.2046 (3), 7.0001 (6), 9.8126 (8)
α, β, γ (°)	100.730 (7), 101.431 (6), 96.098 (6)
*V* (Å^3^)	340.46 (5)
*Z*	1
Radiation type	Mo *K*α
μ (mm^−1^)	1.03
Crystal size (mm)	0.13 × 0.07 × 0.05

Data collection	
Diffractometer	XtaLAB Synergy R, DW system, HyPix
Absorption correction	Multi-scan (*CrysAlis PRO*; Rigaku OD, 2025 ▸)
*T*_min_, *T*_max_	0.698, 1.000
No. of measured, independent and observed [*I* > 2σ(*I*)] reflections	3959, 1563, 1373
*R*_int_	0.036
(sin θ/λ)_max_ (Å^−1^)	0.649

Refinement	
*R*[*F*^2^ > 2σ(*F*^2^)], *wR*(*F*^2^), *S*	0.036, 0.088, 1.05
No. of reflections	1563
No. of parameters	126
H-atom treatment	H atoms treated by a mixture of independent and constrained refinement
Δρ_max_, Δρ_min_ (e Å^−3^)	0.48, −0.36

Computer programs: *CrysAlis PRO* (Rigaku OD, 2025 ▸), *SHELXT* (Sheldrick, 2015*a* ▸), *SHELXL2019/3* (Sheldrick, 2015*b* ▸) and *OLEX2* (Dolomanov *et al.*, 2009 ▸).

## supplementary crystallographic information

### catena-Poly[[tetraaquamanganese(II)]-µ-1,5-dihydroxynaphthalene-\ 2,6-dicarboxylato] Crystal data

**Table:**

[Mn(C_12_H_6_O_6_)(H_2_O)_4_]	*Z* = 1
*M**_r_* = 373.17	*F*(000) = 191
Triclinic, *P*1	*D*_x_ = 1.820 Mg m^−^^3^
*a* = 5.2046 (3) Å	Mo *K*α radiation, λ = 0.71073 Å
*b* = 7.0001 (6) Å	Cell parameters from 2369 reflections
*c* = 9.8126 (8) Å	θ = 3.0–30.5°
α = 100.730 (7)°	µ = 1.03 mm^−^^1^
β = 101.431 (6)°	*T* = 150 K
γ = 96.098 (6)°	Plate, clear yellow
*V* = 340.46 (5) Å^3^	0.13 × 0.07 × 0.05 mm

### catena-Poly[[tetraaquamanganese(II)]-µ-1,5-dihydroxynaphthalene-\ 2,6-dicarboxylato] Data collection

**Table:**

XtaLAB Synergy R, DW system, HyPix diffractometer	1563 independent reflections
Radiation source: Rotating-anode X-ray tube, Rigaku (Mo) X-ray Source	1373 reflections with *I* > 2σ(*I*)
Mirror monochromator	*R*_int_ = 0.036
Detector resolution: 10.0000 pixels mm^-1^	θ_max_ = 27.5°, θ_min_ = 2.2°
ω scans	*h* = −6→6
Absorption correction: multi-scan (CrysAlisPro; Rigaku OD, 2025)	*k* = −9→8
*T*_min_ = 0.698, *T*_max_ = 1.000	*l* = −12→10
3959 measured reflections	

### catena-Poly[[tetraaquamanganese(II)]-µ-1,5-dihydroxynaphthalene-\ 2,6-dicarboxylato] Refinement

**Table:**

Refinement on *F*^2^	Primary atom site location: dual
Least-squares matrix: full	Hydrogen site location: mixed
*R*[*F*^2^ > 2σ(*F*^2^)] = 0.036	H atoms treated by a mixture of independent and constrained refinement
*wR*(*F*^2^) = 0.088	*w* = 1/[σ^2^(*F*_o_^2^) + (0.0456*P*)^2^] where *P* = (*F*_o_^2^ + 2*F*_c_^2^)/3
*S* = 1.05	(Δ/σ)_max_ < 0.001
1563 reflections	Δρ_max_ = 0.48 e Å^−^^3^
126 parameters	Δρ_min_ = −0.35 e Å^−^^3^
0 restraints	

### catena-Poly[[tetraaquamanganese(II)]-µ-1,5-dihydroxynaphthalene-\ 2,6-dicarboxylato] Special details

**Table:**

Geometry. All esds (except the esd in the dihedral angle between two l.s. planes) are estimated using the full covariance matrix. The cell esds are taken into account individually in the estimation of esds in distances, angles and torsion angles; correlations between esds in cell parameters are only used when they are defined by crystal symmetry. An approximate (isotropic) treatment of cell esds is used for estimating esds involving l.s. planes.

### catena-Poly[[tetraaquamanganese(II)]-µ-1,5-dihydroxynaphthalene-\ 2,6-dicarboxylato] Fractional atomic coordinates and isotropic or equivalent isotropic displacement parameters (Å^2^)

**Table:**

	*x*	*y*	*z*	*U*_iso_*/*U*_eq_	
Mn1	0.500000	0.000000	0.500000	0.01475 (16)	
O5	0.7619 (3)	−0.1558 (2)	0.63695 (17)	0.0171 (3)	
O1	0.4392 (3)	0.18483 (19)	0.68599 (15)	0.0165 (3)	
O2	0.5957 (3)	0.4855 (2)	0.66930 (15)	0.0192 (3)	
O3	0.1314 (3)	0.1263 (2)	0.84218 (16)	0.0159 (3)	
O4	0.8266 (3)	0.2198 (2)	0.50602 (19)	0.0255 (4)	
C2	0.2981 (4)	0.4521 (3)	0.8231 (2)	0.0134 (4)	
C4	−0.0062 (4)	0.3969 (3)	0.9786 (2)	0.0132 (4)	
C1	0.4570 (4)	0.3746 (3)	0.7202 (2)	0.0143 (4)	
C6	0.3040 (4)	0.6571 (3)	0.8679 (2)	0.0148 (4)	
H6	0.408213	0.744971	0.830212	0.018*	
C3	0.1441 (4)	0.3249 (3)	0.8791 (2)	0.0121 (4)	
C5	0.1639 (4)	0.7320 (3)	0.9640 (2)	0.0154 (4)	
H5	0.171912	0.870198	0.992500	0.018*	
H4A	0.825 (6)	0.336 (4)	0.557 (3)	0.050 (9)*	
H4B	0.947 (5)	0.218 (4)	0.469 (3)	0.028 (7)*	
H3	0.225 (5)	0.107 (4)	0.788 (3)	0.030 (7)*	
H5A	0.713 (6)	−0.256 (4)	0.659 (3)	0.047 (9)*	
H5B	0.836 (6)	−0.097 (4)	0.703 (3)	0.037 (9)*	

### catena-Poly[[tetraaquamanganese(II)]-µ-1,5-dihydroxynaphthalene-\ 2,6-dicarboxylato] Atomic displacement parameters (Å^2^)

**Table:**

	*U*^11^	*U*^22^	*U*^33^	*U*^12^	*U*^13^	*U*^23^
Mn1	0.0180 (2)	0.0118 (2)	0.0157 (3)	0.00224 (17)	0.00881 (17)	0.00079 (17)
O5	0.0203 (8)	0.0137 (8)	0.0179 (9)	0.0009 (6)	0.0063 (7)	0.0036 (7)
O1	0.0225 (7)	0.0108 (7)	0.0181 (8)	0.0025 (6)	0.0119 (6)	0.0004 (6)
O2	0.0262 (8)	0.0142 (7)	0.0225 (8)	0.0040 (6)	0.0162 (6)	0.0046 (6)
O3	0.0226 (8)	0.0098 (7)	0.0178 (8)	0.0024 (6)	0.0126 (6)	0.0010 (6)
O4	0.0237 (9)	0.0190 (9)	0.0346 (10)	−0.0009 (7)	0.0189 (8)	−0.0030 (7)
C2	0.0167 (9)	0.0121 (9)	0.0121 (10)	0.0038 (7)	0.0056 (7)	0.0009 (7)
C4	0.0151 (9)	0.0135 (10)	0.0120 (10)	0.0034 (8)	0.0048 (7)	0.0028 (7)
C1	0.0177 (9)	0.0136 (10)	0.0107 (10)	0.0027 (8)	0.0025 (8)	0.0005 (7)
C6	0.0187 (10)	0.0118 (9)	0.0149 (10)	0.0012 (8)	0.0059 (8)	0.0041 (8)
C3	0.0167 (9)	0.0085 (9)	0.0102 (9)	0.0029 (7)	0.0019 (7)	0.0006 (7)
C5	0.0213 (10)	0.0090 (9)	0.0170 (10)	0.0037 (8)	0.0065 (8)	0.0023 (8)

### catena-Poly[[tetraaquamanganese(II)]-µ-1,5-dihydroxynaphthalene-\ 2,6-dicarboxylato] Geometric parameters (Å, º)

**Table:**

Mn1—O5	2.2335 (15)	O4—H4A	0.88 (3)
Mn1—O5^i^	2.2335 (15)	O4—H4B	0.79 (3)
Mn1—O1^i^	2.1310 (13)	C2—C1	1.490 (3)
Mn1—O1	2.1310 (13)	C2—C6	1.416 (3)
Mn1—O4	2.1521 (16)	C2—C3	1.394 (3)
Mn1—O4^i^	2.1521 (16)	C4—C4^ii^	1.417 (4)
O5—H5A	0.80 (3)	C4—C3	1.422 (3)
O5—H5B	0.71 (3)	C4—C5^ii^	1.421 (3)
O1—C1	1.297 (2)	C6—H6	0.9500
O2—C1	1.241 (2)	C6—C5	1.365 (3)
O3—C3	1.361 (2)	C5—H5	0.9500
O3—H3	0.79 (3)		
			
O5—Mn1—O5^i^	180.0	Mn1—O4—H4B	132.8 (19)
O1^i^—Mn1—O5^i^	89.70 (5)	H4A—O4—H4B	112 (3)
O1—Mn1—O5^i^	90.30 (5)	C6—C2—C1	120.58 (17)
O1^i^—Mn1—O5	90.30 (5)	C3—C2—C1	120.88 (17)
O1—Mn1—O5	89.70 (5)	C3—C2—C6	118.53 (18)
O1^i^—Mn1—O1	180.0	C4^ii^—C4—C3	118.3 (2)
O1^i^—Mn1—O4	92.89 (6)	C4^ii^—C4—C5^ii^	120.0 (2)
O1—Mn1—O4^i^	92.89 (6)	C5^ii^—C4—C3	121.77 (17)
O1^i^—Mn1—O4^i^	87.11 (6)	O1—C1—C2	116.00 (17)
O1—Mn1—O4	87.11 (6)	O2—C1—O1	122.15 (18)
O4—Mn1—O5^i^	88.22 (6)	O2—C1—C2	121.85 (17)
O4^i^—Mn1—O5	88.22 (6)	C2—C6—H6	119.1
O4—Mn1—O5	91.78 (6)	C5—C6—C2	121.73 (18)
O4^i^—Mn1—O5^i^	91.78 (6)	C5—C6—H6	119.1
O4—Mn1—O4^i^	180.0	O3—C3—C2	121.77 (17)
Mn1—O5—H5A	124 (2)	O3—C3—C4	116.76 (17)
Mn1—O5—H5B	116 (2)	C2—C3—C4	121.47 (17)
H5A—O5—H5B	102 (3)	C4^ii^—C5—H5	120.0
C1—O1—Mn1	130.36 (12)	C6—C5—C4^ii^	120.04 (18)
C3—O3—H3	106.0 (18)	C6—C5—H5	120.0
Mn1—O4—H4A	115 (2)		
			
Mn1—O1—C1—O2	−24.2 (3)	C6—C2—C1—O2	−1.0 (3)
Mn1—O1—C1—C2	155.01 (13)	C6—C2—C3—O3	−179.21 (17)
C2—C6—C5—C4^ii^	−0.2 (3)	C6—C2—C3—C4	0.4 (3)
C4^ii^—C4—C3—O3	178.9 (2)	C3—C2—C1—O1	0.9 (3)
C4^ii^—C4—C3—C2	−0.7 (3)	C3—C2—C1—O2	−179.87 (18)
C1—C2—C6—C5	−178.81 (19)	C3—C2—C6—C5	0.1 (3)
C1—C2—C3—O3	−0.3 (3)	C5^ii^—C4—C3—O3	−0.5 (3)
C1—C2—C3—C4	179.27 (17)	C5^ii^—C4—C3—C2	179.88 (18)
C6—C2—C1—O1	179.77 (17)		

Symmetry codes: (i) −*x*+1, −*y*, −*z*+1; (ii) −*x*, −*y*+1, −*z*+2.

### catena-Poly[[tetraaquamanganese(II)]-µ-1,5-dihydroxynaphthalene-\ 2,6-dicarboxylato] Hydrogen-bond geometry (Å, º)

**Table:**

*D*—H···*A*	*D*—H	H···*A*	*D*···*A*	*D*—H···*A*
O4—H4*A*···O2	0.88 (3)	2.02 (3)	2.756 (2)	141 (3)
O4—H4*B*···O5^iii^	0.79 (3)	2.04 (3)	2.814 (2)	169 (3)
O3—H3···O1	0.79 (3)	1.75 (3)	2.480 (2)	153 (3)
O5—H5*A*···O2^iv^	0.80 (3)	1.88 (3)	2.664 (2)	168 (3)
O5—H5*B*···O3^v^	0.71 (3)	2.13 (3)	2.798 (2)	157 (3)

Symmetry codes: (iii) −*x*+2, −*y*, −*z*+1; (iv) *x*, *y*−1, *z*; (v) *x*+1, *y*, *z*.
