# Supplementary material for: Crystal structure of catena-poly[[tetra­aquamangan­ese(II)]-μ-1,5-dihy­droxynaphthalene-2,6-di­carboxyl­ato]
Source: Acta Crystallogr E Crystallogr Commun. 2026 Jan 20;82(Pt 2):178–81. doi: 10.1107/S205698902600040X (PMC12874242; doi:10.1107/S205698902600040X)
Supplement: Supplementary file 4 [file e-82-00178-sup3.pdf]

Table S1 comparison of selected bond distances.

	M-O1 (carboxy)	M-O4 (H_2O)	M-O5 (H_2O)
Mn	2.1310(13)	2.1521(16)	2.2335(15)
Co	2.0750 (9)	2.0931 (10)	2.1023 (10)

Table S2 comparison of selected bond angles.

	O1(carboxy)-M-O4(H_2O)	O1(carboxy)-M-O5(H_2O)	O4(H_2O)-M-O5(H_2O)
Mn	87.11(6)	89.70(5)	91.78(6)
Co	89.40(4)	88.91(4)	91.72(4)

Table S3 comparison of intra-molecular hydrogen bonds.

M	D—H···A	D—H	H···A	D···A	D—H···A
Mn	O(phenol)—H···O(calboxy)	0.79(3)	1.75(3)	2.480(2)	153(3)
Co	O(phenol)—H···O(calboxy)	0.78 (2)	1.81 (2)	2.5168 (13)	149.1 (19)

Table S4 comparison of inter-molecular hydrogen bonds.

M	D—H···A	D—H	H···A	D···A	D—H···A
Mn	O(H_2O)—H···O(calboxy)	0.80(3)	1.88(3)	2.664(2)	168(3)
Co	O(H_2O)—H···O(calboxy)	0.78 (2)	1.96 (2)	2.7284 (15)	168 (2)

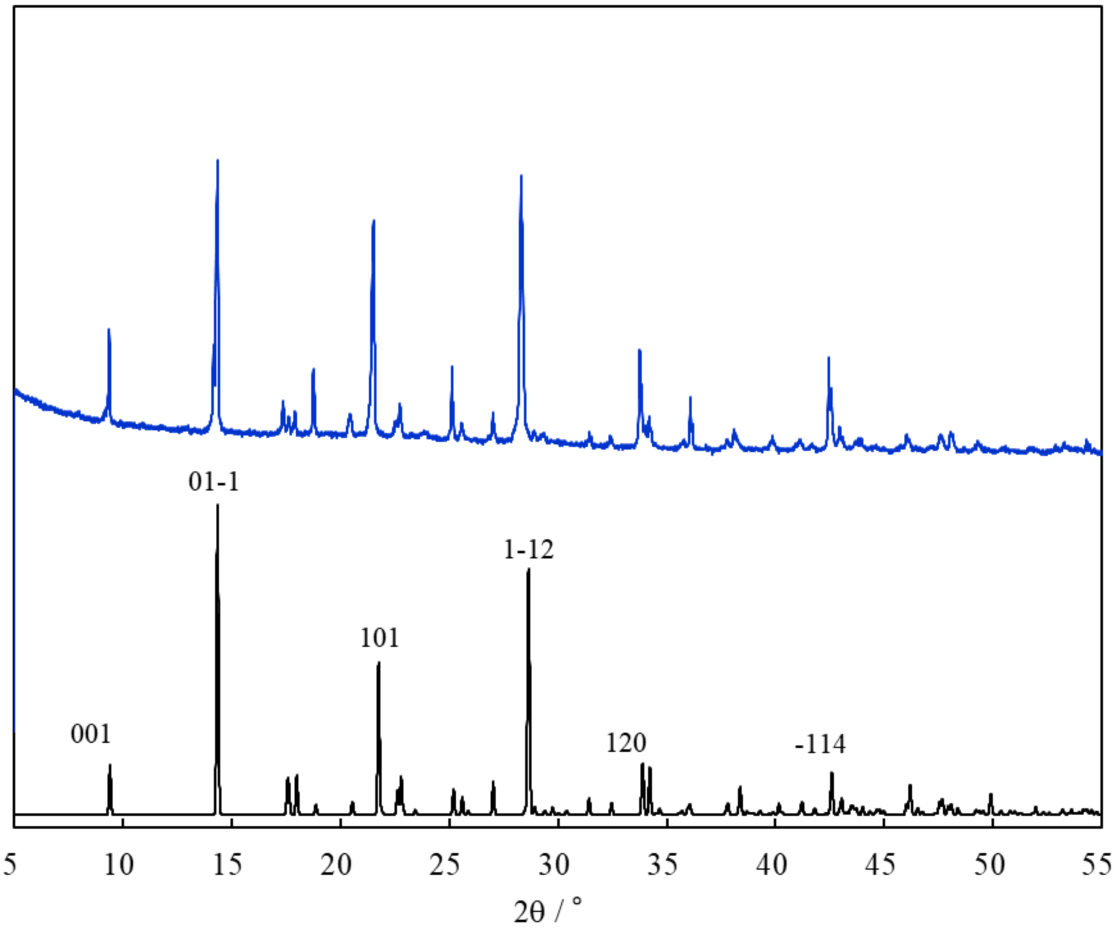

Figure S1 Calculated (black) and simulated (blue) PXRD patterns for the $[Mn(H_2dondc)(H_2O)_4]_n$.
